# Are femoroacetabular impingement tomographic angles associated with the histological assessment of labral tears? A cadaveric study

**DOI:** 10.1371/journal.pone.0199352

**Published:** 2018-06-21

**Authors:** Leandro Ejnisman, Benjamin G. Domb, Felipe Souza, Consuelo Junqueira, Jose Ricardo Negreiros Vicente, Alberto Tesconi Croci

**Affiliations:** 1 Instituto de Ortopedia e Traumatologia, Hospital das Clinicas HCFMUSP, Faculdade de Medicina, Universidade de Sao Paulo, Sao Paulo, Brazil; 2 American Hip Institute, Westmont, Illinois, USA Hinsdale Orthopedics, Westmont, Illinois, United States of America; Rush University Medical Center, UNITED STATES

## Abstract

**Purpose:**

This study sought to investigate the association between tomographic femoroacetabular impingement (FAI) angles and histologically evaluated labral tears. The authors hypothesized that cadavers presenting with cam and pincer morphologies would present a higher prevalence of acetabular labral tears.

**Methods:**

Twenty fresh cadavers were submitted to computed tomography. Standard FAI angles were measured, including the alpha angle, femoral version, acetabular version, Tonnis angle and center-edge angle. A cam lesion was defined as an alpha angle greater than 50^o^. A pincer lesion was defined as a center-edge angle greater than 40^o^, a Tonnis angle less than 0^o^ or acetabular version less than 0^o^. After dissection, three fragments of each acetabulum, corresponding to the antero-superior, superior and postero-superior acetabular rim, were obtained. These fragments were submitted to routine histological preparation. Each slide was evaluated for possible labral tears. Tears were classified according to their Seldes type.

**Results:**

The mean age of the cadavers was 50.2 years (SD: 7.4; 13 males). Sixteen (80%) of the cadavers had a cam lesion, and eight cadavers (40%) had a pincer lesion. Histologically, 16 (80%) of the cadavers had a labral tear in at least one region. According to the Seldes classification, 60.7% and 28.6% of these labral tears were type 1 and type 2, respectively. A mixed type of labral tear (10.7%), which represented a new form of Seldes tear, was described. Cadavers with a labral tear had significantly higher alpha angles than other cadavers (53.29^o^ *vs* 49.33^o^, *p* = 0.01). Pincer lesions were not associated with labral tears. We found no association between pincer or cam lesions and Seldes classification.

**Conclusion:**

Cadavers presenting with higher alpha angles had a higher incidence of labral tears. No association was found between FAI and Seldes classification.

**Clinical relevance:**

This study demonstrated a high prevalence of FAI abnormalities associated with histological alterations in a cadaveric sample. Joint damage may be present in the early stages of FAI.

## Introduction

Femoroacetabular impingement (FAI) is characterized by abnormal contact between the proximal femur and the acetabulum [1,2]. It is a common cause of hip pain and early hip osteoarthritis. Although the literature includes an abundance of articles that describe clinical results after the surgical treatment of this condition, few publications have addressed basic scientific knowledge regarding FAI.

Asymptomatic individuals can present with high rates of radiographic abnormalities in the hip. A recent systematic review reported a 37% prevalence of cam deformity and a 67% prevalence of pincer deformity in 2,114 asymptomatic hips [3]. Moreover, a labral tear prevalence of 68.1% was found. Hystollogical evaluation of the labrum in FAI patients demonstrated a chronic degenerative process without a significant inflammatory component [4]. The labra were thickened with a disorganized and cystic matrix. However, it is not known whether the asymptomatic population that presents with radiographic abnormalities also presents histological alterations.

The goal of this study was to evaluate the association of tomographic FAI findings with the histological assessment of labral tears in cadavers. The authors hypothesized that cadavers presenting with cam and pincer morphologies would present a higher prevalence of acetabular labral tears.

## Methods

This study was approved by the institutional review board. No funding was received for this project. The inclusion criteria were age between 18 and 60 years old and an absence of lower limb deformities or scars in the hip region. The exclusion criteria were advanced osteoarthritis demonstrated on the computed tomography (CT) or after dissection and iatrogenic lesions to the labrum found during dissection. Cadavers originated from the autopsy department of our institution. Specimens were sequentially added as available for dissection. None of the donors were from a vulnerable population. The local ethics committee waived the need for consent given by the donor or their next of kin.

CT scans were obtained for all cadavers and included a pelvic CT and a knee CT from the side being studied (all scans were performed with the cadaver in the same position). The CT images were saved as DICOM ("Digital Imaging and Communications in Medicine") files and exported to Osirix software (Version 6.0.2, Geneva, Switzerland).

### Tomographic measurement

All angles were measured by a fellowship-trained musculoskeletal radiologist who was blinded to the histological findings. The alpha angle was measured as described by Nötzli et al. [5]. An axial oblique femoral cut was made via cuts that were parallel to the femoral neck and passed through the center of the femoral head. A circle with the approximate diameter of the femoral head was drawn. The alpha angle comprises a line through the center of the femoral head and the center of the femoral neck and a line through the center of the femoral head and the point where the femoral head exits the drawn circle (i.e., the point where the femoral head loses its sphericity) (S1 Fig). The supplemental material contains figures demonstrating all tomographic measurements. A cam deformity was considered when the alpha angle was greater than 50^o^ [6,7].

Femoral anteversion was measured using the technique described by Tomczak et al [8]. A line containing the most posterior aspect of both femoral condyles on the axial view was obtained. A second line containing the center of the femoral head and the center of the femoral neck was considered the femoral neck axis. Femoral version was measured by superimposing both views and measuring the angle between the 2 lines (S2 Fig). Positive angles were considered anteversion, and negative angles were considered retroversion.

Before measuring the acetabular-side angles, the pelvic position was standardized to a neutral pelvic tilt and inclination (anterior pelvic plane alignment) [9,10]. Acetabular version was measured on the axial view, on the superior region of the acetabuli. The version angle comprised a line joining the anterior and posterior bony margins of the acetabulum and a horizontal line joining the posterior margins of both acetabuli (S4 Fig). Positive angles were considered anteversion, and negative angles were considered retroversion.

The coronal image that corresponded to the center of the acetabulum in the axial view was used to measure the lateral center-edge angle (LCEA) and the Tonnis angle (TA). The LCEA comprised a vertical line from the center of the femoral head to the lateral-most point of the acetabulum (S3 Fig). The TA was measured between the horizontal line and a line from the medial portion of the weight-bearing zone to the superior lateral edge of the acetabulum (S5 Fig). Pincer morphology was defined as follows [11]: LCEA≥40^o^ [12] or TA≤0^o^ [13] or acetabular retroversion (AV≤0^o^ with LCEA≥30^o^) [14].

### Histological analysis

**Fig 1:**
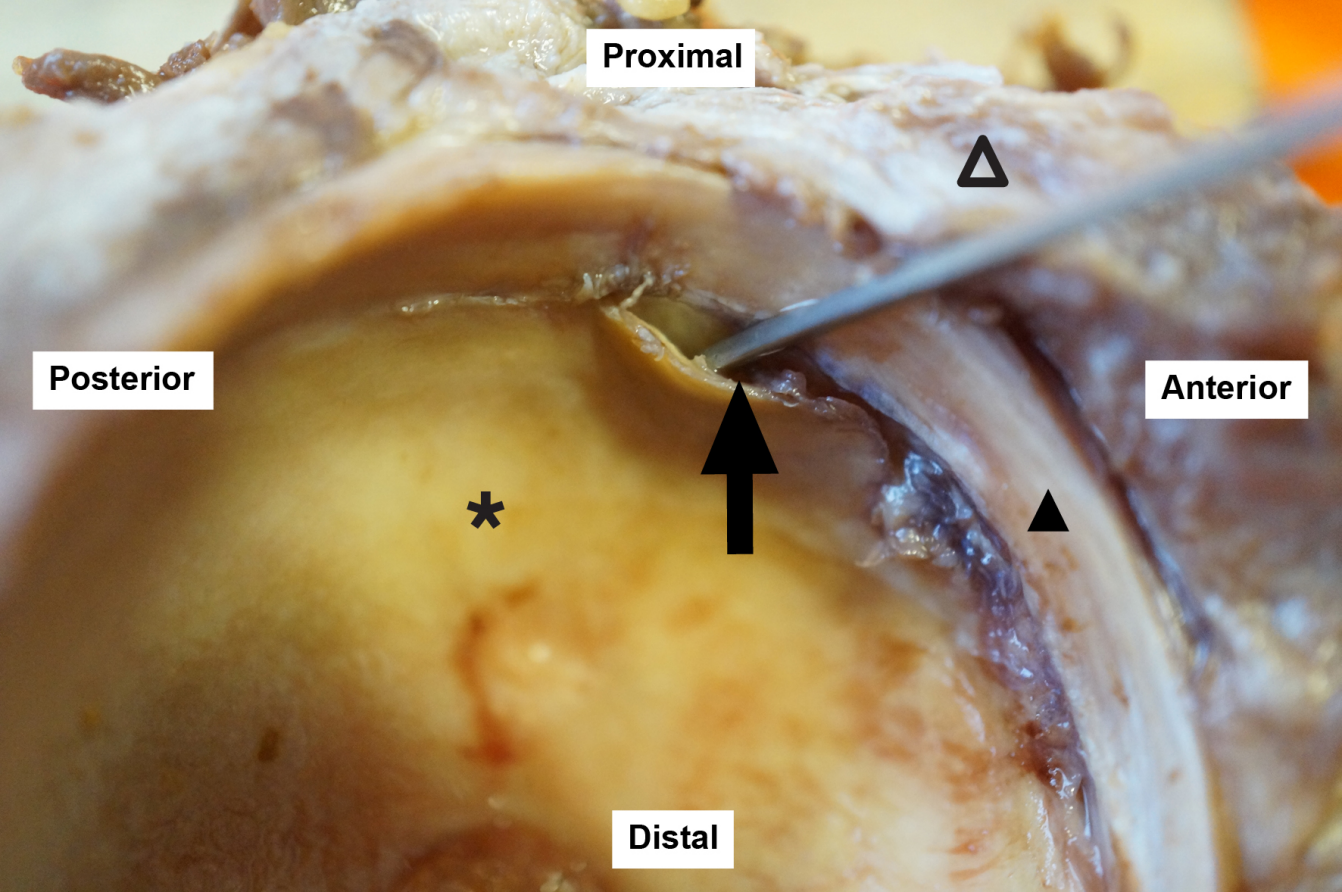
Macroscopic view of an antero-superior labral tear associated with carpet delamination of the acetabular cartilage. Black triangle: acetabular labrum; empty triangle: capsule; black star: acetabular cartilage; black arrow: probe demonstrating chondrolabral separation.

**Fig 2:**
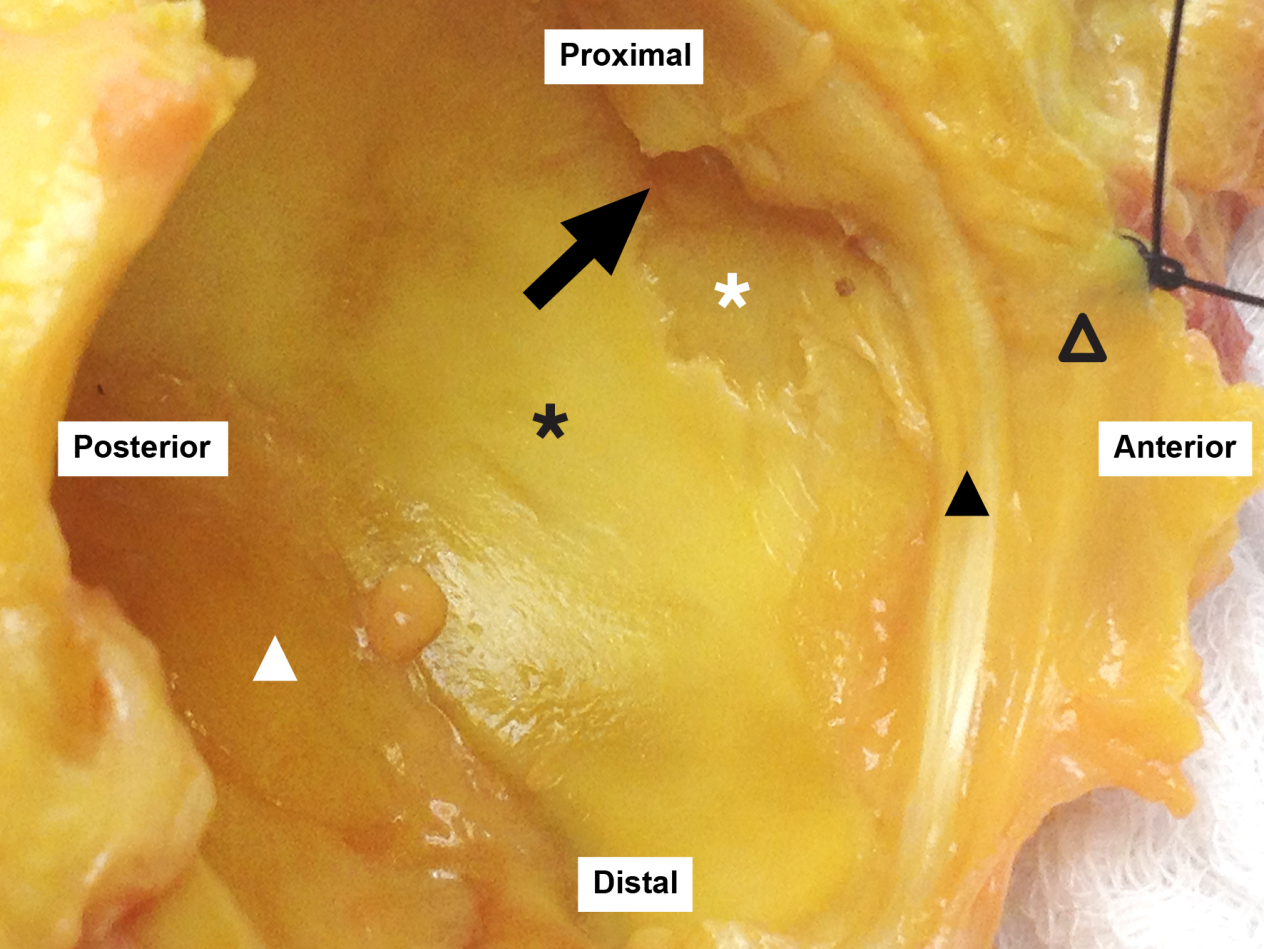
Macroscopic view of a labral tear associated with full-thickness cartilage loss. Black triangle: acetabular labrum; empty triangle: capsule; black star: acetabular cartilage; white star: cartilage damage with bone exposure; black arrow: labral tear; white triangle: pulvinar.

**Fig 3:**
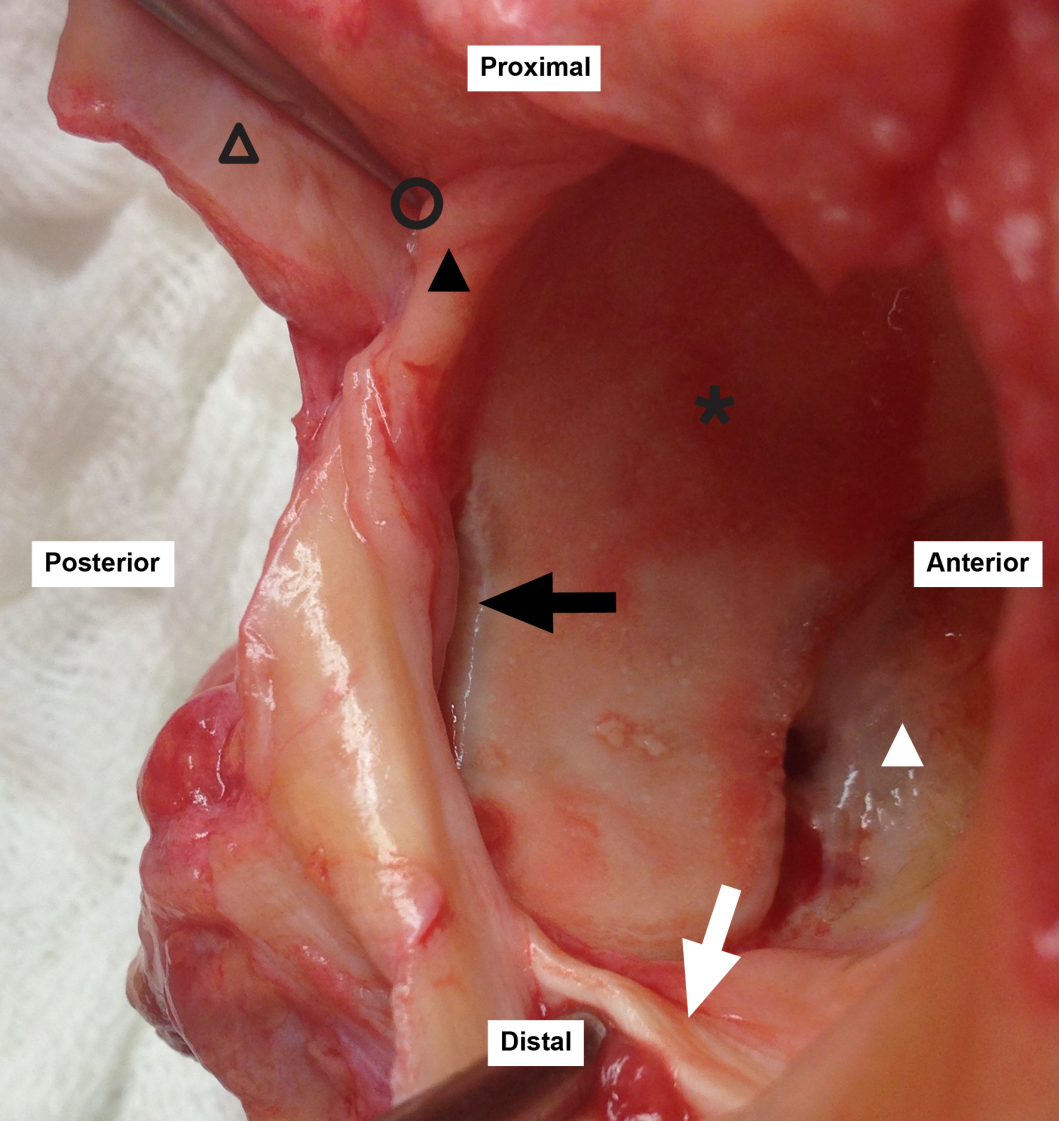
Sublabral sulcus. Black triangle: acetabular labrum; white triangle: pulvinar; empty triangle: capsule; black star: acetabular cartilage; white arrow: transverse ligament; black arrow: sublabral sulcus; black circle: hemostat in the capsule-labral recess.

The cadavers were dissected after CT examination. The acetabulum was obtained via a triple osteotomy using an anterior approach. A macroscopic evaluation was then performed, paying special attention to labral tears, macroscopic chondral damage and sublabral sulci (Figs 1–3).

**Fig 4:**
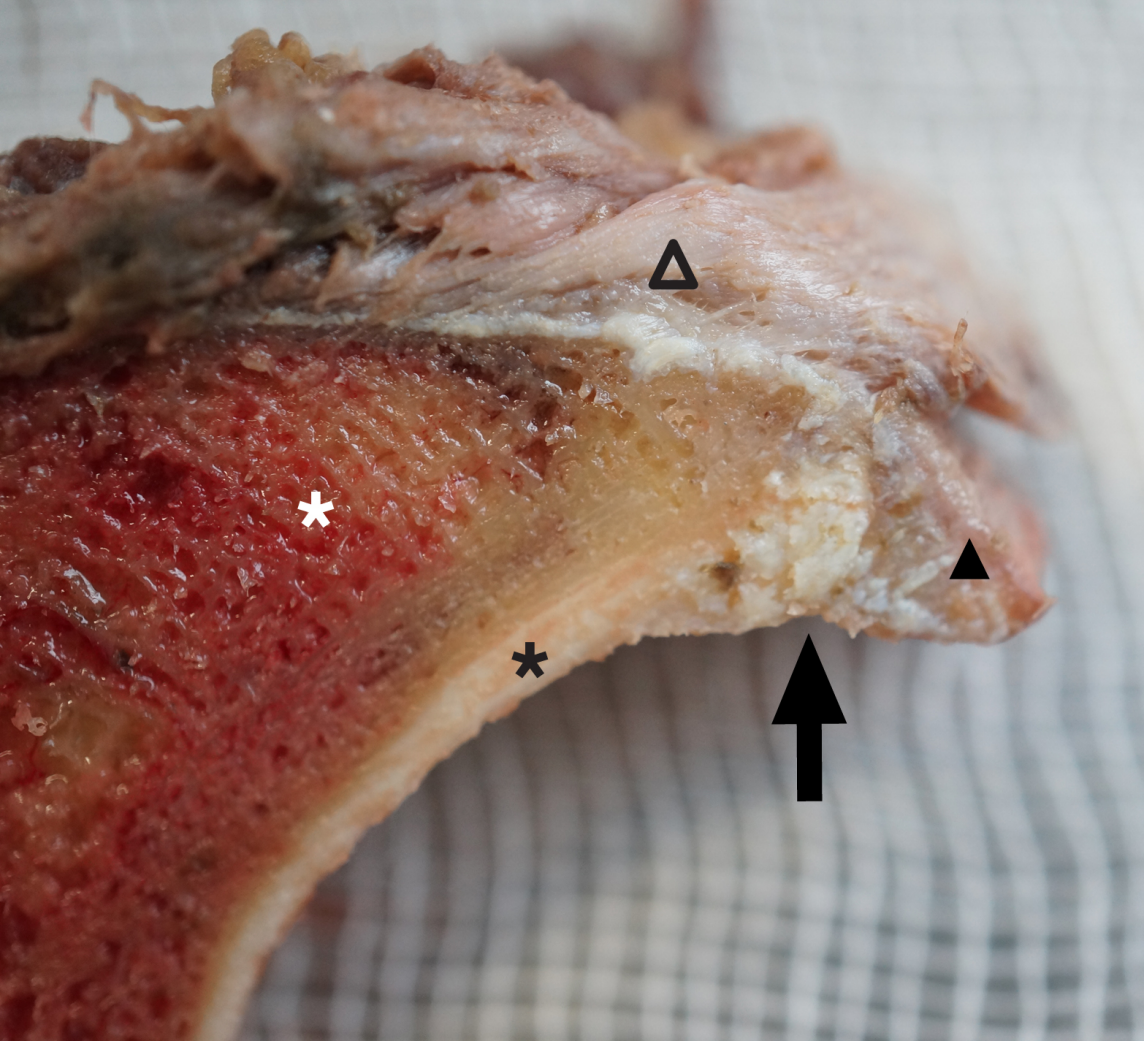
Example of a superior fragment. Black triangle: acetabular labrum; empty triangle: capsule; black star: acetabular cartilage; white star: acetabular bone; black arrow: intact chondrolabral junction.

**Fig 5:**
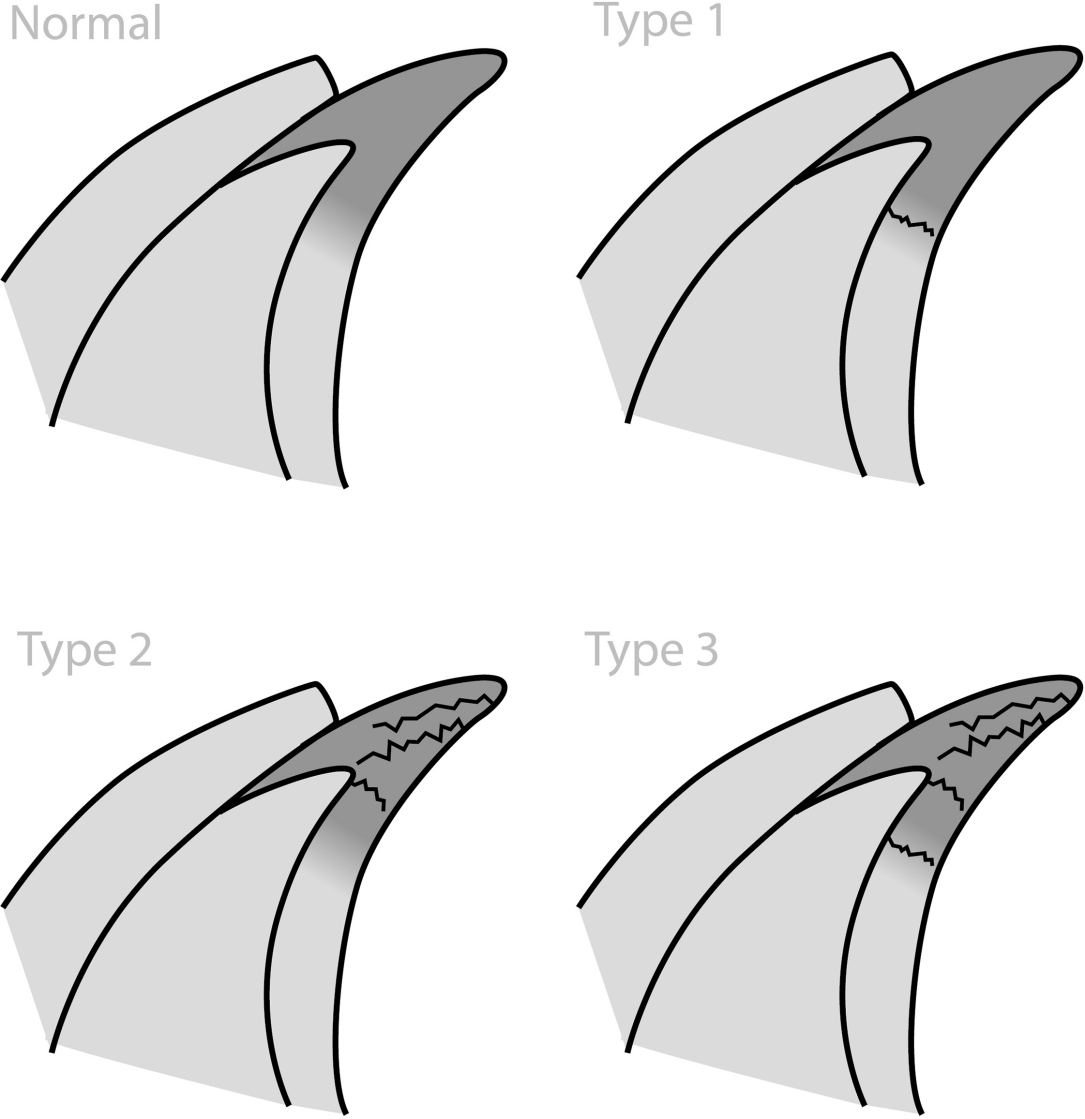
Illustration of the modified Seldes classification.

The acetabuli were fixed in formalin, decalcified with nitric acid and then cut in the radial direction. Three fragments (Fig 4) containing a cross-section of the labrum, capsule, articular cartilage and subchondral bone were obtained from each acetabulum. The fragments were cut from different positions based on the face of a clock [15]: 10, 12 and 2 hours, corresponding to the postero-superior, superior and antero-superior regions, respectively. The fragments were embedded in paraffin, and 5-μm sections were obtained. All slides were stained with hematoxylin and eosin, and Masson’s trichrome. Histological evaluations were performed by an experienced pathologist who was blinded to the tomographic and macroscopic findings. The slides were scrutinized for labral tears; when tears were present, they were categorized using the Seldes classification (Fig 5) [16]. According to Seldes, a type 1 tear consists of the detachment of the labrum from the articular cartilage surface; a type 2 tear consists of one or more cleavage planes of variable depth within the substance of the labrum. The original classification was modified to include a type 3 tear, which is a mixed tear with characteristics of both type 1 and type 2 tears.

### Statistical methods

Continuous variables (age, height, weight, BMI, alpha angle, femoral version, LCEA, acetabular version and TA) were described using means, standard deviations and ranges. The Kolmogorov-Smirnov test was used to test the normality of study data. For the continuous variables of age and tomographic angles, comparisons were performed using t-tests or the Mann-Whitney test, depending on the normality of the data. Correlations of 2 continuous variables (tomographic angles) were determined using Pearson’s correlation coefficient. Radiographic angles were analyzed as categorical data (pincer, cam or mixed), and category frequencies were described. Histological data were also analyzed as categorical data. Categorical variables were compared using the chi-square test or Fisher’s exact test. All reported *p* values were 2-tailed, and an alpha level of 0.05 indicated statistical significance. Statistical analyses were performed using SPSS version 22.0 for Mac statistical software (SPSS, Inc., Chicago, Illinois).

## Results

### Demographics

Initially, one cadaver was studied and considered as a pilot for the feasibility of this project. This pilot was not included in the analysis. Twenty-one fresh frozen cadavers were included in this study. One cadaver was excluded because during dissection, an iatrogenic fracture of the acetabulum occurred with a subsequent labral tear. The demographics of the twenty included specimens are presented in Table 1, and in more details in S1 Table.

**Table 1:** Demographics.

	Mean (standard deviation)	Range
Age (years)	50.2 (7.4)	34–58
Sex (percentage)	70% males	
Height (cm)	167.7 (9.8)	150–187
Weight (kg)	63.6 (10.6)	37.2–77.0
BMI (kg/m)^2^	22.7 (4.1)	15.9–29.6

### Tomographic analysis

The results of the tomographic analysis are described in Table 2. When the subjects were divided by sex, the LCEA was greater in females than in males (41.7^o^ *vs* 33.1^o^, *p* = 0.02) No differences between groups were found for other tomographic angles when the subjects were categorized by sex or by height (*p*>0.05).

**Table 2:** Tomographic angles.

	Mean (standard deviation)	Range
Alpha (^o^)	52.5 (3.1)	45.9–58.3
Center-edge (^o^)	35.7 (7.9)	22.6–51.2
Acetabular version (^o^)	14.1 (5.6)	3.7–23.2
Tonnis (^o^)	6.1 (3.6)	0–15.0
Femoral version (^o^)	12.1 (9.9)	-8.0–30.0

When analyzing FAI types, we found 16 (80%) cadavers that were considered cam positive (males: 85.7% *vs* females: 66.7%, *p*>0.05). Eight (40%) of the specimens were pincer positive (males: 21.4^o^% *vs* females: 83.3%, *p* = 0.01). Six (30%) cadavers were cam and pincer positive and thus were considered mixed type. Thus, 10 (50%) cases had an isolated cam lesion, and 2 (10%) had an isolated pincer lesion.

### Histological analysis

**Fig 6:**
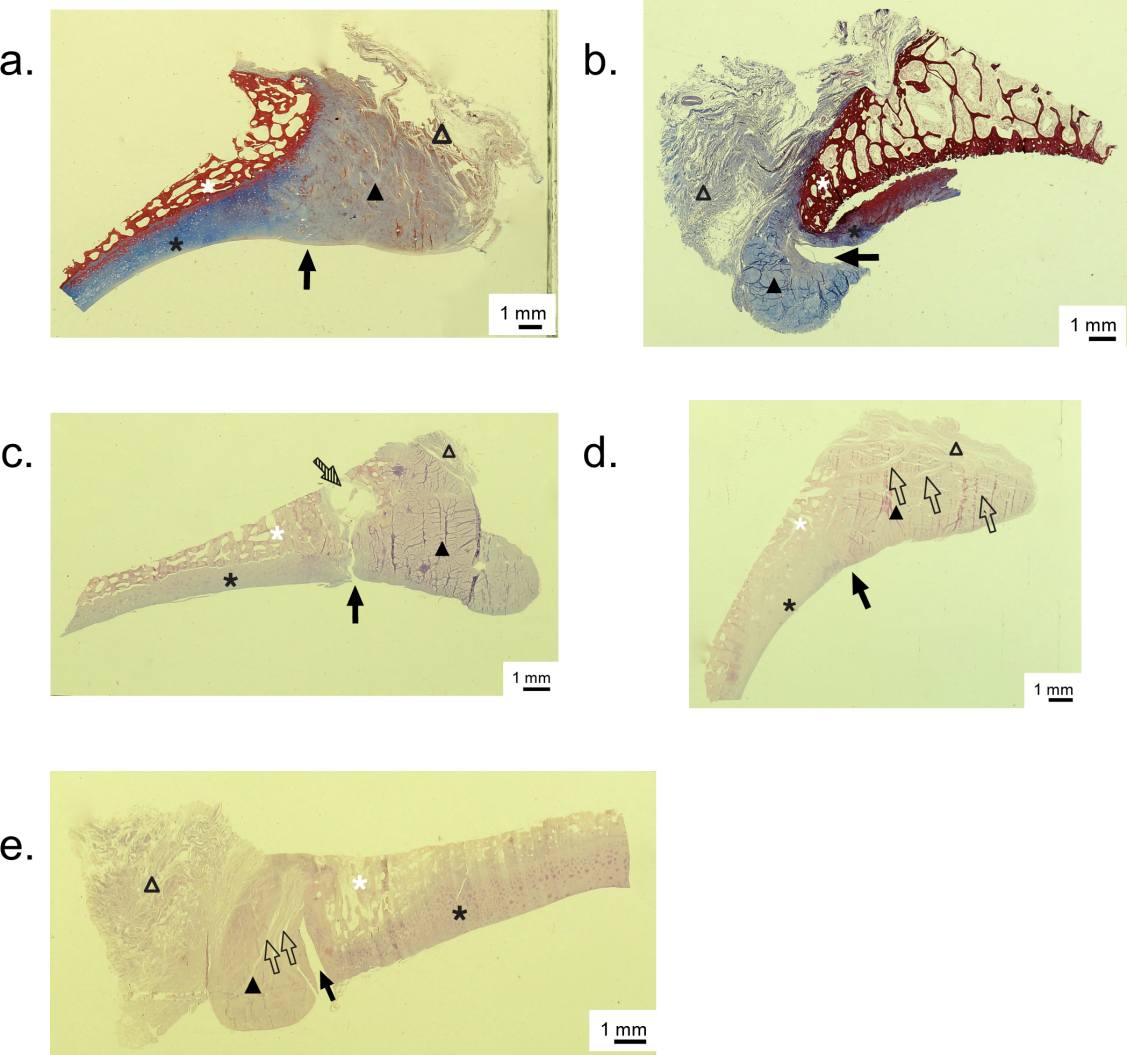
Histological slides. (a) Normal acetabular labrum (Masson’s trichrome stained). (b) Example of a type 1 labral tear associated with cartilage detachment from the acetabular bone (Masson’s trichrome stained). (c) Example of a type 1 labral tear associated with acetabular bone cyst formation (hematoxylin and eosin stained). (d) Example of a type 2 labral tear (hematoxylin and eosin stained). (e) Example of a type 3 labral tear (hematoxylin and eosin stained). Black triangle: acetabular labrum; empty triangle: capsule; black star: acetabular cartilage; white star: acetabular bone; black arrow: chondrolabral junction; striped arrow: acetabular bone cyst; empty arrows: cleavage planes in the labral substance.

After acetabular dissection, 10 (50%) cases presented a macroscopic labral tear. Three (15%) cases had a posterior labral sulcus. Three (15%) specimens presented acetabular chondral damage with visible subchondral bone, and all of these presented a macroscopic labral tear associated with the chondral damage. Microscopically, 16 (80%) specimens presented a labral tear at least in one region (Fig 6). There was no age difference between the cadavers presenting an intact labrum and those with labral tears (no tear: 54 ± 2.4 years old *vs* tear: 49 ± 7.9 years old, *p*>0.05). When divided by region, 65% of the cases presented lesions in the anterior labra, 50% presented lesions in the superior labra, and 25% presented lesions in the posterior labra; these differences were statistically significant (*p* = 0.04). According to the modified Seldes classification, 60.7% of the labral tears were type 1, 28.6% were type 2, and 10.7% were type 3. When analyzing the posterior labrum, of the 3 cases presenting with a posterior sulcus, only one presented a labral tear (Seldes 1). No agreement was found between the macroscopic and microscopic assessment of the labral tears (p>0.05).

### Relationship between tomographic angles and histology

Cadavers presenting with a microscopic labral tear demonstrated higher alpha angles (no tear: 49.3^o^ *vs* tear: 53.3^o^, *p* = 0.01). Additionally, cam-positive individuals presented a higher prevalence of labral tears (*p* = 0.01). No association was found between femoral version, LCEA, TA and acetabular version and the presence of microscopic labral tears (*p*>0.05). No association was found between pincer impingement and labral tears (*p*>0.05). No association was found between FAI type (cam or pincer) and Seldes classification (*p*>0.05).

## Discussion

Our results demonstrate a higher prevalence of labral tears in cadavers with alpha angles higher than 50^o^. This relationship has been previously demonstrated in FAI patients. Johnston et al. [17] reported an association between the alpha angle and detachment of the base of the labrum observed during hip arthroscopy. Additionally, studies have demonstrated that hips with an alpha angle of 65^o^ or more are at increased risk of substantial cartilage damage observed intra-operatively [18]. The same association between alterations of the proximal femur and the acetabular labrum was also reported in asymptomatic populations. In an MRI study of 45 volunteers, individuals with an osseous bump in the head/neck junction were more likely to present a labral tear [19].

The location of the alpha angle measurement is controversial. As described by Notzli et al. [5] the alpha angle is measured in the axial oblique slice, which corresponds to the anterior part of the femur. However, some authors propose that multiple axial cuts should be used to evaluate the alpha angle in different positions of the femur because the antero-superior portion of the femoral head/neck junction can present greater deformity [20–22]. In a series of 41 patients, the mean oblique axial plane and mean maximal radial alpha angles were 53.4^o^ and 70.5^o^, respectively [21]. In the current study, we opted to measure the alpha angle in the axial oblique cut because we believe that method is still routinely used by most hip surgeons and more closely resembles routine radiological evaluation.

Furthermore, the normal values of the alpha angle are debatable. The literature presents different thresholds for differentiating normal and abnormal, with recommendations ranging from 42^o^ to 63^o^ [5,23–25]. We defined cam deformity as an alpha angle greater than 50^o^ [6,7]. We used this value to investigate whether even lower alpha angles would influence labral damage. When evaluating the association between the alpha angle and the presence of labral tears, we found a positive association when the alpha angle was analyzed as a continuous variable. This analysis is independent of the cam deformity cut-off, which decreases the importance of its value in the current study.

No association between pincer FAI and labral tears or between femoral version and labral tears was found. It is important to keep in mind that more than the bony anatomy plays a role in labral tearing. Hip instability and psoas impingement also cause labral damage; however, these factors were not investigated in this study [26,27]. Moreover, alterations other than mechanical abnormalities, such as increased inflammation and neovascularization, have been demonstrated in FAI patients [28,29]. The role of each of these components in the pathophysiology of labral injury remains to be established.

Ito et al. [4] evaluated acetabular labra obtained from symptomatic FAI patients. The labra demonstrated a chronic degenerative process without an inflammatory component. Moreover, the labra were hypercelular, cointained larger rounded fibroblasts and lacked typical perpendicullar collagen bundles. Interestingly, the authors did not find an association between FAI type and histopathologic change, which we could not also demonstrate in our study. When comparing the hystollogical findings in labral tears in asymptomatic cadavers and symptomatic FAI patients, one cannot find a distinguishing difference in the hystollogical presentation. Therefore, FAI diagnosis cannot rely exclusively on imaging. Recently, the term FAI syndrome was coined, consisting of a triad of symptoms, clinical signs and imaging findings [30].

Although no relationship between age and labral tears was found, one may exist. We found a 75% prevalence of labral tears in a population with a mean age of 50 years. Two previous studies investigated the prevalence of labral tears in cadavers. Seldes et al. [16] found a prevalence of 96% (mean age 78 years), while Vawkland and Letwanich [31] found a prevalence of 66% (mean age 55 years). It is interesting to note that the study that included older patients presented a higher prevalence of labral tears.

We found no correlation between the macroscopic and microscopic assessment of labral tears. There were specimens in which a macroscopic labral tear was found, with no corresponding microscopic tear. A possible explanation is that histological slides in these specimens were from a different anatomical position than the macroscopic tear, because acetabular fragments were obtained from pre-established anatomical positions regardless of the presence of a macroscopic tear. In other specimens a microscopic tear was found where no macroscopic tear was previously observed. We speculate these cases represent minor tears, which were not visible on a macroscopic inspection. This finding might explain patients presenting with clinical findings of a symptomatic labral tear, but with no labral alterations on imaging studies an/or normal labra during arthroscopic procedure.

Beck et al. [1] reported the results of a macroscopic evaluation of the relationship of FAI type and the pattern of acetabular damage. Cam impingement caused an antero-superior separation between the healthy labrum and the cartilage, while pincer impingement caused a circumferential degeneration of the labrum. Khol et al. [32] also demonstrated the influence of FAI type on the histology of damaged acetabular cartilage. The findings of Beck et al. and Khol et al. could be extrapolated, and the Seldes classification could have an association with the FAI type. If such an association exists, a mixed type of histological labral tear would be expected. Therefore, our finding of a third type of Seldes classification was not surprising. Nevertheless, our study did not find an association between FAI type and Seldes classification. It is important to note that Beck et al. and Kohl et al. analyzed patients with isolated cam and pincer impingement. The high prevalence of different hip abnormalities in our population made it difficult to isolate the effect of each alteration. On top of that, both Beck et al. and Kohl et al. studied FAI patients, whereas our study examined cadavers, which might explain the different findings.

We recognize certain limitations of this study. The clinical history of the cadavers was not recorded. We assumed that they were asymptomatic, but this hypothesis was not confirmed. It would be interesting to assess their clinical history to determine whether any of the individuals with labral tears and chondral damage were indeed symptom free. Moreover, the habits of the studied population were not known. FAI radiographic abnormalities are more frequent in the athletic population [3], so it would be interesting to evaluate the sports habits of our sample. Another limitation is the number of specimens evaluated. The power of this study could be improved with a bigger sample. However, histological studies are cumbersome, and our sample is consistent with those of previous papers on labral histology [32,33].

## Conclusions

This study found a high prevalence of FAI morphological abnormalities and labral tears in cadavers. A modification of the Seldes classification was described that included a mixed type tear. Cadavers presenting with higher alpha angles had a higher incidence of labral tears. No association was found between FAI and Seldes classification.

## Supporting information

S1 Fig Alpha angle measurement. (A) The axial oblique femoral plane is determined by line “a” which passes trough the center of the femoral head and the center of the femoral neck. (B) The alpha angle comprises a line “b” trough the center of the femoral head and the center of the femoral neck, and a line “c” trough the center of the femoral head and the point where the femoral head exists the drawn circle “d”.
(JPG)

S2 Fig Femoral version measurement. A femoral cut containing the center of the femoral neck, and a knee cut containing the posterior portion of the femoral condyles are superimposed. The femoral version angle is comprised by a line “a” containing the most posterior part of both femoral condyles and a line “b”containing the center of the femoral neck.
(TIF)

S3 Fig Center-edge angle measurement. Line “a”comprises both femoral head centers, and determines the horizontal plane. Circle “d”determines the center of the femoral head. Line “b”is perpendicular to line “a” and passes trough the center of the femoral head. Line “c”passes trough the center of the femoral head and the most lateral aspect of the acetabulum. The center-edge angle is determined by lines “b” and “c”.
(TIF)

S4 Fig Acetabular version measurement. Line “a”is determined by the most posterior part of both acetabulum, line “c” comprises the anterior and posterior acetabular walls and line “b”is perpendicular to line “a”. Acetabular version is determined by lines “b” and “c”.
(TIF)

S5 Fig Tönnis angle measurement. Line “a”determines the horizontal plane (parallel to a line comprising both femoral head centers) and containing the most medial aspect of the acetabular sourcil. Line “b”connects the most medial and the most lateral aspects of the acetabular sourcil The Tönnis angle is determined by line “a”and “b”.
(TIF)

S1 Table Specimens’characteristics. (XLSX)
