# Supplementary figures and images for: Are femoroacetabular impingement tomographic angles associated with the histological assessment of labral tears? A cadaveric study
Source: PLoS One. 2018 Jun 21;13(6):e0199352. doi: 10.1371/journal.pone.0199352 (PMC6013197; doi:10.1371/journal.pone.0199352)

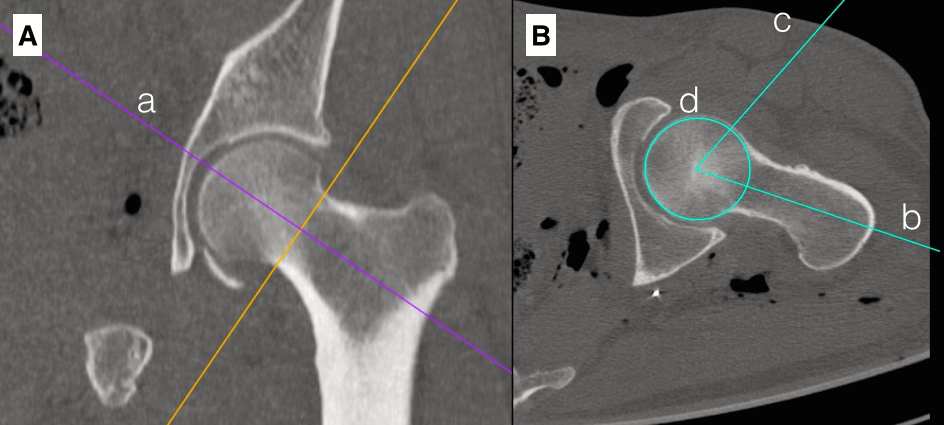

Supplement: S1 Fig — (A) The axial oblique femoral plane is determined by line “a” which passes trough the center of the femoral head and the center of the femoral neck. (B) The alpha angle comprises a line “b” trough the center of the femoral head and the center of the femoral neck, and a line “c” trough the center of the femoral head and the point where the femoral head exists the drawn circle “d”. (JPG) [file pone.0199352.s001.jpg]

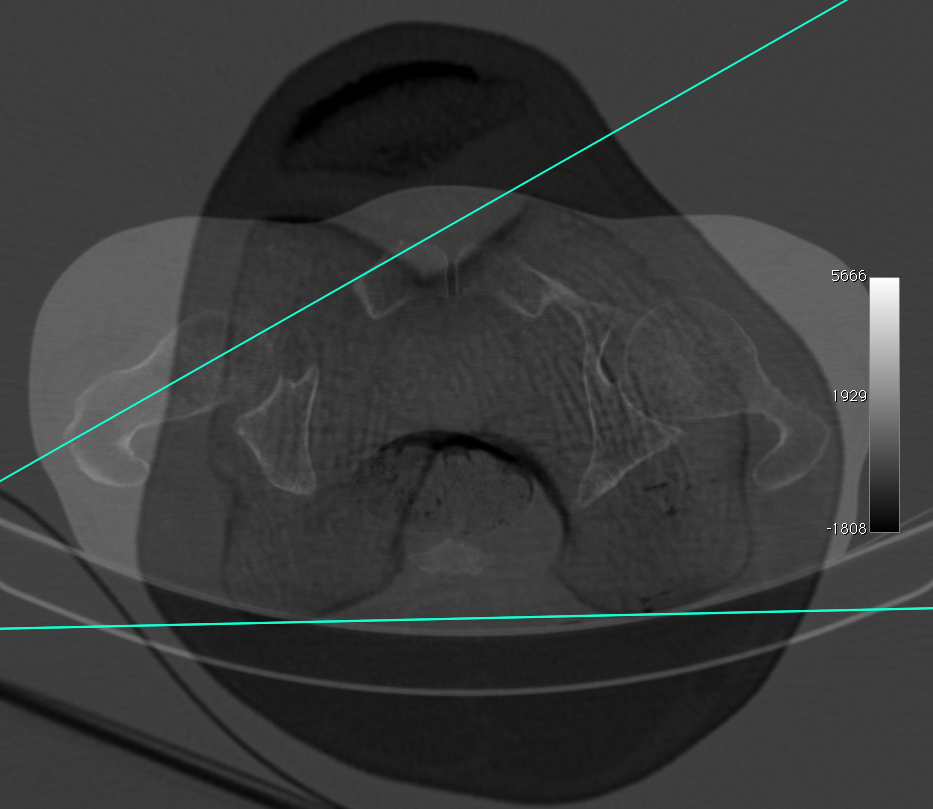

Supplement: S2 Fig — A femoral cut containing the center of the femoral neck, and a knee cut containing the posterior portion of the femoral condyles are superimposed. The femoral version angle is comprised by a line “a” containing the most posterior part of both femoral condyles and a line “b”containing the center of the femoral neck. (TIF) [file pone.0199352.s002.tif]

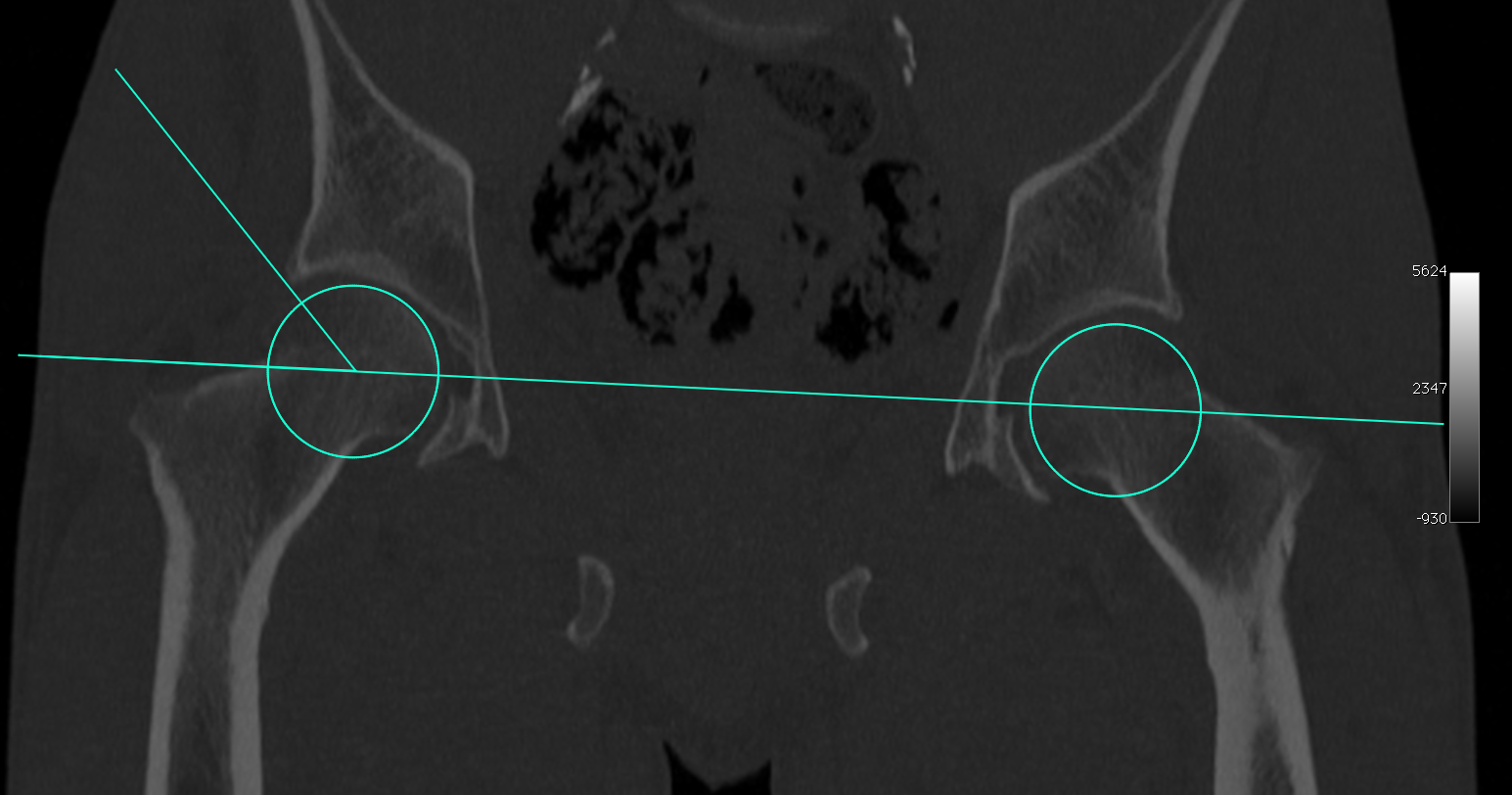

Supplement: S3 Fig — Line “a”comprises both femoral head centers, and determines the horizontal plane. Circle “d”determines the center of the femoral head. Line “b”is perpendicular to line “a” and passes trough the center of the femoral head. Line “c”passes trough the center of the femoral head and the most lateral aspect of the acetabulum. The center-edge angle is determined by lines “b” and “c”. (TIF) [file pone.0199352.s003.tif]

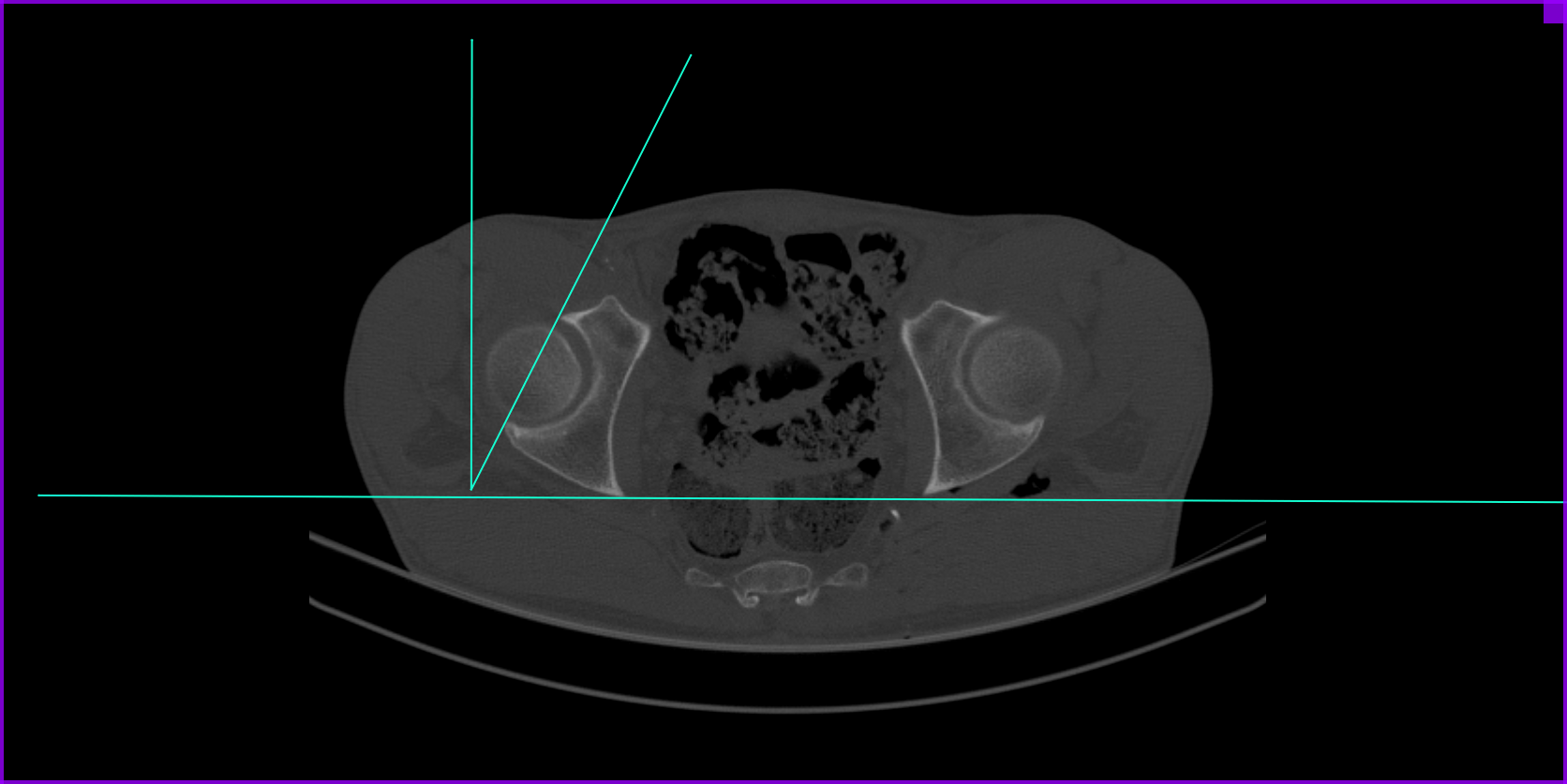

Supplement: S4 Fig — Line “a”is determined by the most posterior part of both acetabulum, line “c” comprises the anterior and posterior acetabular walls and line “b”is perpendicular to line “a”. Acetabular version is determined by lines “b” and “c”. (TIF) [file pone.0199352.s004.tif]

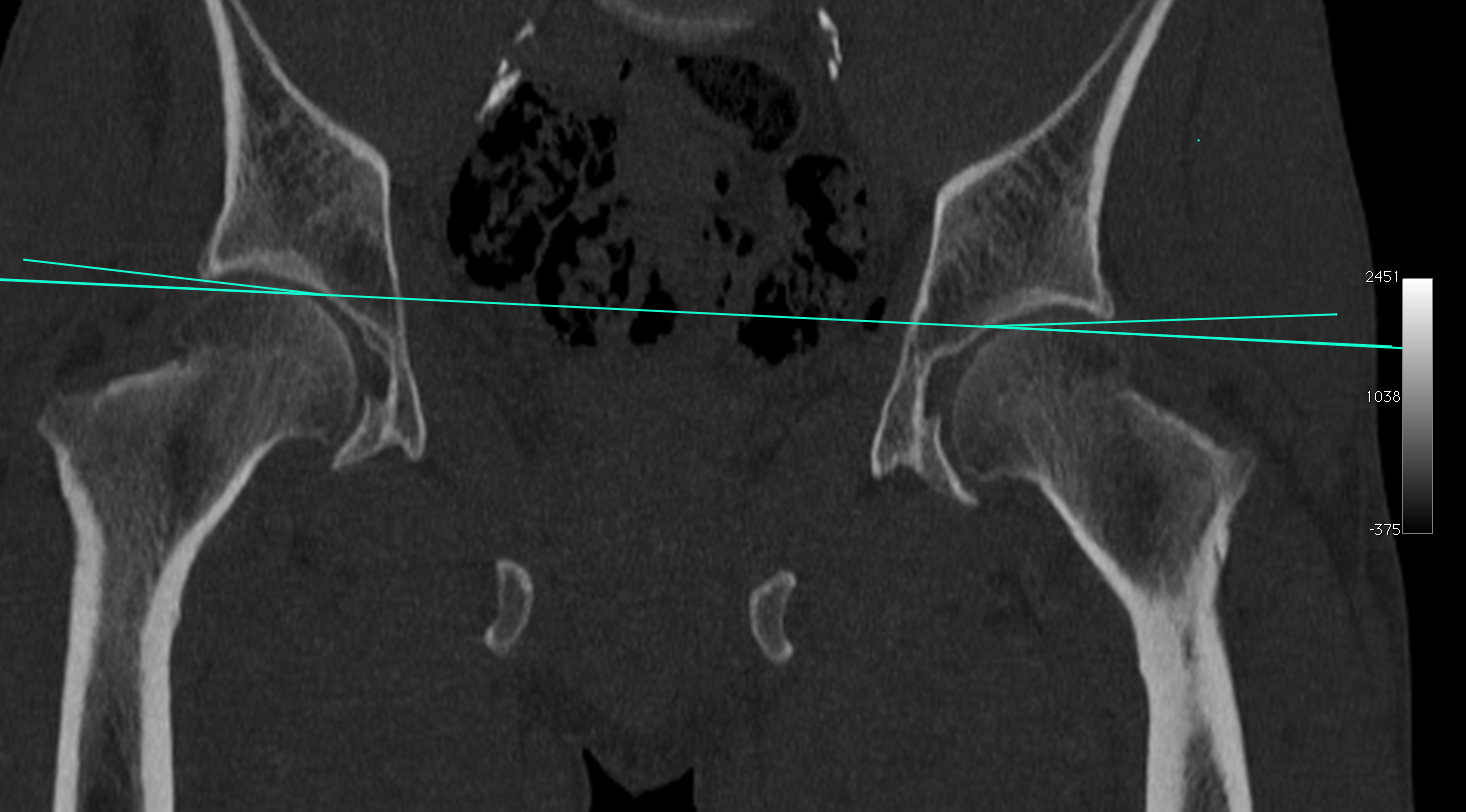

Supplement: S5 Fig — Line “a”determines the horizontal plane (parallel to a line comprising both femoral head centers) and containing the most medial aspect of the acetabular sourcil. Line “b”connects the most medial and the most lateral aspects of the acetabular sourcil The Tönnis angle is determined by line “a”and “b”. (TIF) [file pone.0199352.s005.tif]
